# Associations of serum amyloid A and 25‐hydroxyvitamin D with diabetic nephropathy: A cross‐sectional study

**DOI:** 10.1002/jcla.24283

**Published:** 2022-02-08

**Authors:** Qian Liu, Jin Sun, Tongdao Xu, Guangrong Bian, Fumeng Yang

**Affiliations:** ^1^ Department of Laboratory Medicine The Second People's Hospital of Lianyungang Lianyungang China; ^2^ Department of Endocrinology The Second People's Hospital of Lianyungang Lianyungang China

**Keywords:** 25‐hydroxyvitamin D, diabetic nephropathy, serum amyloid A, type 2 diabetes mellitus

## Abstract

**Background:**

The present study investigated the relationships between serum amyloid A (SAA), 25‐hydroxyvitamin D (25(OH)VD) and diabetic nephropathy (DN) to provide evidence for the prevention and management of DN.

**Methods:**

A total of 182 patients with type 2 diabetes mellitus (T2DM) were enrolled in this study. The levels of SAA, 25(OH)VD, and other conventional indicators were measured and analyzed. Receiver operating characteristic curve analysis was applied for the combined measurement of SAA and 25(OH)VD, and risk factors for DN were evaluated using binary logistic regression analysis.

**Results:**

The levels of SAA in T2DM patients were significantly higher than those in healthy subjects, and the level significantly increased with the progression of DN (*p* < 0.05). In contrast, the level of 25(OH)VD in T2DM patients was significantly lower than that in healthy subjects, and the level significantly decreased with the progression of DN (*p* < 0.05). The combined measurement of SAA and 25(OH)VD distinguished DN patients from T2DM patients better than the measurement of SAA or 25(OH)VD alone. SAA was an independent risk factor for DN, and 25(OH)VD was an independent protective factor for DN.

**Conclusion:**

SAA and 25(OH)VD might be used as potential markers to identify patients at increased risk of developing DN.

## Graphical abstract

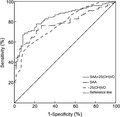

## 1 INTRODUCTION

Due to the increasing incidence of type 2 diabetes mellitus (T2DM), it has become an important problem affecting public health.^1^, ^2^ Diabetic nephropathy (DN), one of the common long‐term microvascular complications of T2DM, is the main cause of chronic kidney disease and end‐stage renal disease.^3^ The early features of DN are glomerular and tubular epithelial hypertrophy, thickened basement membrane and excessive extracellular matrix deposition, which, in turn, leads to glomerular sclerosis and tubular interstitial fibrosis.^4^ The main clinical manifestations of DN are progressive proteinuria and a decline in renal function.^4^ At present, the mechanism of DN is not completely clear, but its early diagnosis and timely treatment are critical to reversing or delaying the renal function damage, so the identification of serum markers is very important.

Serum amyloid A (SAA) is an acute‐phase response protein.^5^ When inflammation occurs, the expression of SAA in the liver, fat, and kidney increases significantly.^6^, ^7^ SAA in the kidney is secreted by glomerular cells, including podocytes and mesangial cells. Its main role is to promote the expression of inflammatory cytokines in fibroblasts, macrophages and podocytes.^6^, ^7^ Previous studies have reported that SAA is closely related to the occurrence and progression of DN.^8^, ^9^ Serum 25‐hydroxyvitamin D (25(OH)VD) is a metabolite of vitamin D in the body, is relatively stable and is considered to be the best indicator of vitamin D levels in the body.^10^ Previous studies have reported a close relationship between vitamin D and T2DM, and there is evidence that vitamin D deficiency leads to the occurrence of diabetes.^11^, ^12^, ^13^, ^14^ Recent reports primarily focused on the clinical application of independent indicators (SAA or [25(OH)VD]) in diabetes and its microvascular diseases, but there are no clinical applications of the combined detection of SAA and (25(OH)VD) in DN.^15^, ^16^, ^17^ Therefore, we investigated the relationships between SAA, 25(OH)VD and DN in a cross‐sectional study to provide evidence for the prevention and management of DN.

## 2 MATERIALS AND METHODS

### 2.1 Subjects

This cross‐sectional study was conducted at the Department of Endocrinology and Laboratory Medicine, the Second People's Hospital of Lianyungang, from August 2020 to February 2021. Subjects with T2DM were diagnosed by using the American Diabetes Association criteria.^18^ The following exclusion criteria were used: (1) type 1 diabetes or other special types of diabetes; (2) acute or chronic inflammation‐related diseases; (3) urinary system, respiratory system, digestive system, and cardiovascular and cerebrovascular diseases; (4) kidney diseases not caused by diabetes; (5) malignant tumors; (6) body mass index (BMI) <18.5 kg/m^2^; (7) uncontrolled blood pressure (SBP >180 mmHg and/or DBP >120 mmHg); (8) estimated glomerular filtration rate (eGFR) <60 ml/min/1.73 m^2^; (9) non‐diabetic proteinuria or albuminuria; and (10) recently received medical or surgical treatment.

**FIGURE 1:**
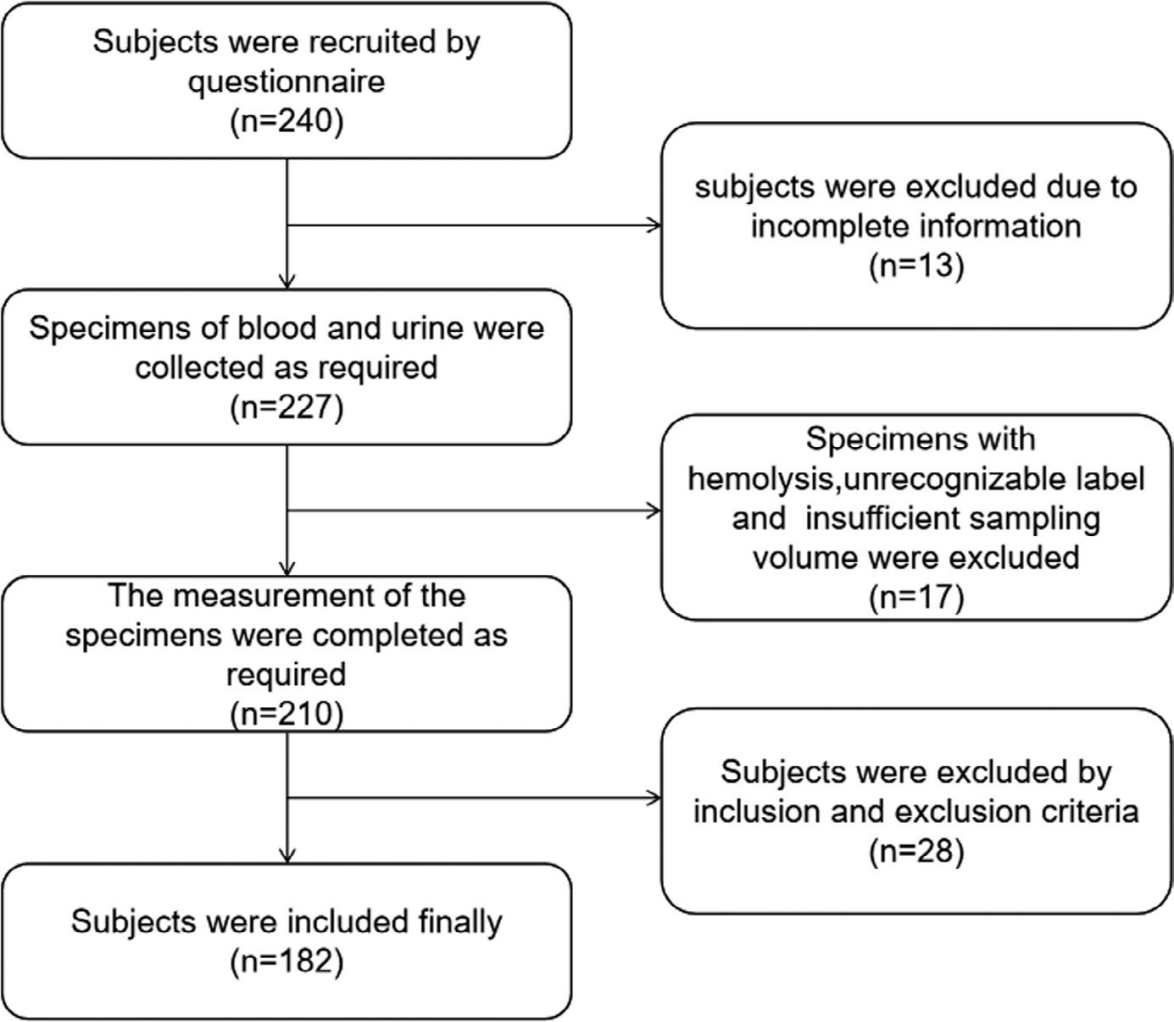
CONSORT flow diagram of participants with T2DM

A total of 240 patients with T2DM participated in this study, but only 182 patients were included in the end (58 patients were excluded based on the exclusion criteria). The selection process of subjects with T2DM are shown in Figure 1. In addition, based on the urinary albumin‐to‐creatinine ratio (UACR), patients with T2DM were divided into three groups^17^: (1) the normal albuminuria group (NA group, UACR <30 mg/g, *n* = 60), (2) the microalbuminuria group (MA group, 30 ≤ UACR < 300, *n* = 63), and (3) the clinical albuminuria group (CP group, UACR ≥300, *n* = 59). Consistent with the American Diabetes Association guidelines,^19^ individuals in the MA and CP groups were defined as having DN (UACR ≥30 mg/g) in this study. A total of 180 sex‐ and age‐matched healthy individuals with normal fasting blood glucose who received physical examinations in the Department of Physical Examination Center were recruited as the control group. This study was approved by the Ethics Committee of the Second People's Hospital of Lianyungang (2020X021), and all subjects signed an informed consent form.

### 2.2 Anthropometric and laboratory measurement

General information on sex, age, smoking status, course of disease and medical records was obtained from the questionnaire. Height and weight were measured, and body mass index (BMI) was calculated as weight (kg) divided by the square of height (m^2^). Blood pressure was measured as the mean of two results measured in a sitting position, and the interval between the two measurements was at least 15 min.

All subjects fasted for 8–10 h, and venous blood samples were collected from 6 AM to 8 AM. EDTA K_2_ anticoagulated venous blood (2 ml) was used for the detection of glycated hemoglobin A1c (HbA1c), and venous blood without anticoagulant (5 ml, centrifugal parameters: 1200 *g* for 10 min) was used for the detection of serum urea (Urea), creatinine (Crea), total cholesterol (TC), triglycerides (TG), high‐density lipoprotein cholesterol (HDL‐C), low‐density lipoprotein cholesterol (LDL‐C), fasting blood glucose (FBG), SAA and 25(OH)VD. Moreover, random spot urine specimens (10 ml, centrifugal parameters: 400 *g* for 10 min) were collected for the detection of urinary albumin and Crea, and then the UACR results were calculated. The estimated glomerular filtration rate (eGFR) was calculated with the Cockcroft‐Gault equation: eGFR_CG_ = [(140‐age) × weight (kg)]/SCr × 72 × [0.85 if female] and adjusted for a body surface area of 1.73 m^2^.^20^ All assays were conducted within 6 h.

HbA1c levels were measured using high‐performance liquid chromatography (HLC‐723G8 glycosylated hemoglobin analyzer, Tosoh). The concentrations of Urea, Crea, TC, TG and FBG were measured via enzymatic methods (AU5800 biochemical analyzer, Beckman Coulter). The levels of HDL‐C and LDL‐C were measured using selective clearance methods (AU5800 Biochemical Analyzer, Beckman Coulter). The levels of SAA and urinary albumin were detected using immunoturbidimetry (AU5800 biochemical analyzer, Beckman Coulter). The 25(OH)VD level was measured using the liquid chromatography tandem mass spectrometry method (API 3200 mass spectrometer, Sciex). The minimum detection level for SAA was 0.1 mg/L, and its intra‐ and inter‐assay coefficients of variation were 5% and 10%, respectively. The minimum detection level for 25(OH)VD was 1.0 ng/ml, and its intra‐ and inter‐assay coefficients of variation were 4% and 6%, respectively.

Before specimen detection, the detection systems were calibrated and maintained. The internal quality control met the analytical performance, and external quality assessments were qualified.

### 2.3 Statistical analyses

Statistical analyses were performed using IBM SPSS Version 19 (IBM Corp.). The Kolmogorov‐Smirnov test was used to analyze the normality of quantitative variables, and normally distributed data were expressed as the means and standard deviations. Unpaired t tests and chi‐square tests were used for comparisons between groups with quantitative and categorical variables, respectively. One‐way analysis of variance was used to compare the means among multiple groups, and the LSD test was used for pairwise comparisons. The combined detection of SAA and 25(OH)VD was analyzed by using receiver operating characteristic (ROC) curves, and risk factors for DN were evaluated using binary logistic regression analysis. Two‐tailed *p*‐values less than 0.05 were considered statistically significant.

## 3 RESULTS

### 3.1 Characteristics of patients with T2DM

This study included a total of 182 patients with T2DM and 180 healthy subjects (matched for sex and age). The age, sex, BMI, blood pressure (SBP and DBP), smoking status, duration of T2DM, Urea, Crea, and other laboratory indicators of T2DM and control subjects are listed in Table 1. There were no differences in the means of BMI, Urea, Crea, TC, TG, HDL‐C, or LDL‐C in either group (*p* > 0.05). The levels of blood pressure (SBP and DBP), FBG, HbA1c, UACR, and SAA were significantly higher in the T2DM group than in the control group (*p* < 0.05). In addition, the levels of eGFR and 25(OH)VD were significantly lower in the T2DM group than in the control group (*p* < 0.05).

**TABLE 1:** Conventional and laboratory characteristics of all subjects

Indicators	Control group (*n* = 180) *n* = 30	T2DM group (*n* = 182)	*t* test/χ^2^	*p*‐value
Age (years)	53.82 ± 6.59	55.07 ± 7.83	1.63	0.1032
Sex (Females/males)	89/91	105/77	2.16	0.1421
BMI (kg/m^2^)	24.25 ± 1.45	24.55 ± 1.57	1.88	0.0605
SBP (mmHg)	130.70 ± 5.36	137.90 ± 11.78	7.50	<0.0001
DBP (mmHg)	79.66 ± 3.86	80.75 ± 4.55	2.47	0.0139
Smoking status (yes/no)	62/118	78/104	2.36	0.1247
Duration of T2DM (years)	–	14.69 ± 5.65	–	–
Urea (mmol/L)	5.95 ± 1.61	6.18 ± 1.98	1.14	0.2536
Crea (µmol/L)	73.45 ± 11.80	76.30 ± 17.10	1.85	0.0658
TC (mmol/L)	4.94 ± 0.32	4.99 ± 0.43	1.50	0.1353
TG (mmol/L)	1.03 ± 0.29	1.11 ± 0.40	1.81	0.0710
HDL‐C (mmol/L)	1.11 ± 0.10	1.11 ± 0.11	1.92	0.0567
LDL‐C (mmol/L)	3.11 ± 0.26	3.18 ± 0.38	1.88	0.0608
FBG (mmol/L)	5.53 ± 0.40	10.53 ± 2.21	29.87	<0.0001
HbA1c (%)	5.46 ± 0.35	7.81 ± 0.79	36.60	<0.0001
eGFR (ml/min/1.73m^2^)	102.30 ± 7.79	98.29 ± 8.34	4.73	<0.0001
UACR (mg/g)	18.15 ± 3.89	164.10 ± 81.39	16.91	<0.0001
SAA (mg/L)	8.38 ± 2.47	25.97 ± 9.08	15.45	<0.0001
25(OH)VD (ng/ml)	32.43 ± 7.37	25.76 ± 6.53	9.12	<0.0001

Note

Sex and smoking status indicators are expressed in numbers, and other indicators are expressed as means and standard deviations. Chi‐square tests were used for sex and smoking status indicators, and unpaired *t* tests were used for the other indicators. *p*‐values <0.05 were considered statistically significant.

Abbreviations: 25(OH)VD, 25‐hydroxyvitamin D; BMI, body mass index; Crea, creatinine; DBP, diastolic blood pressure; eGFR, estimated glomerular filtration rate; FBG, fasting blood glucose; HbA1c, glycated hemoglobin A1c; HDL‐C, high‐density lipoprotein cholesterol; LDL‐C, low‐density lipoprotein cholesterol; SAA, serum amyloid A; SBP, systolic blood pressure; TC, total cholesterol; TG, triglycerides; UACR, urinary albumin‐to‐creatinine ratio; Urea, urea.

### 3.2 Levels of SAA and 25(OH)VD among T2DM patients grouped by UACR

**FIGURE 2:**
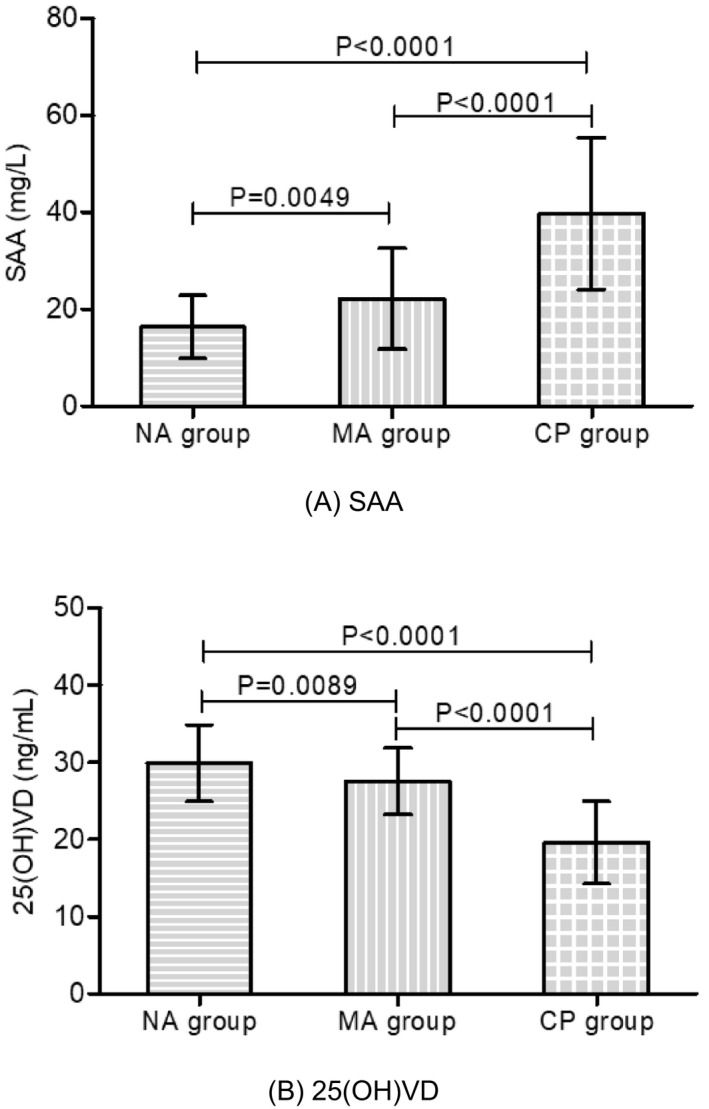
Comparison of SAA and 25(OH)VD among patients with T2DM. Bar graph showing the levels of SAA (A) and 25(OH)VD (B) among the NA group, MA group and CP group. Error bars in the bar graph indicate the mean and standard deviation

According to the levels of UACR, patients with T2DM were divided into three groups (NA group, MA group and CP group). Table 2 and Figure 2 show the levels of SAA and 25(OH)VD in subgroups of T2DM patients, which indicated that both SAA and 25(OH)D were significantly different in the subgroups of T2DM patients (*p* < 0.05). Even with the different levels of UACR, both SAA and 25(OH)VD showed significant differences (*p* < 0.05). The level of SAA significantly increased with the progression of DN, but the level of 25(OH)VD significantly decreased with the progression of DN.

**TABLE 2:** Levels of SAA and 25(OH)VD among T2DM grouped by UACR

Indicators	T2DM			*F*‐value	*p*‐value
	NA group (UACR <30, *n* = 60)	MA group (30 ≤ UACR < 300, *n* = 63)	CP group (UACR ≥300, *n* = 59)		
SAA (mg/L)	16.34 ± 6.52	22.22 ± 9.81	39.77 ± 15.64	67.52	<0.0001
25(OH)VD (ng/ml)	29.90 ± 4.96	27.57 ± 4.32	19.61 ± 5.34	72.81	<0.0001

Note

SAA and 25(OH)VD indicators are expressed as means and standard deviations. One‐way analysis of variance was used to compare means (SAA and 25(OH)VD) between the NA, MA and CP groups. *p*‐values <0.05 were considered statistically significant.

Abbreviations: 25(OH)VD, 25‐hydroxyvitamin D; CP group, clinical proteinuria group; MA group, microalbuminuria group; NA group, normal albuminuria group; SAA, serum amyloid A; T2DM, type 2 diabetes mellitus; UACR, urinary albumin‐to‐creatinine ratio.

### 3.3 Combined measurement of SAA and 25(OH)VD in patients with DN

**FIGURE 3:**
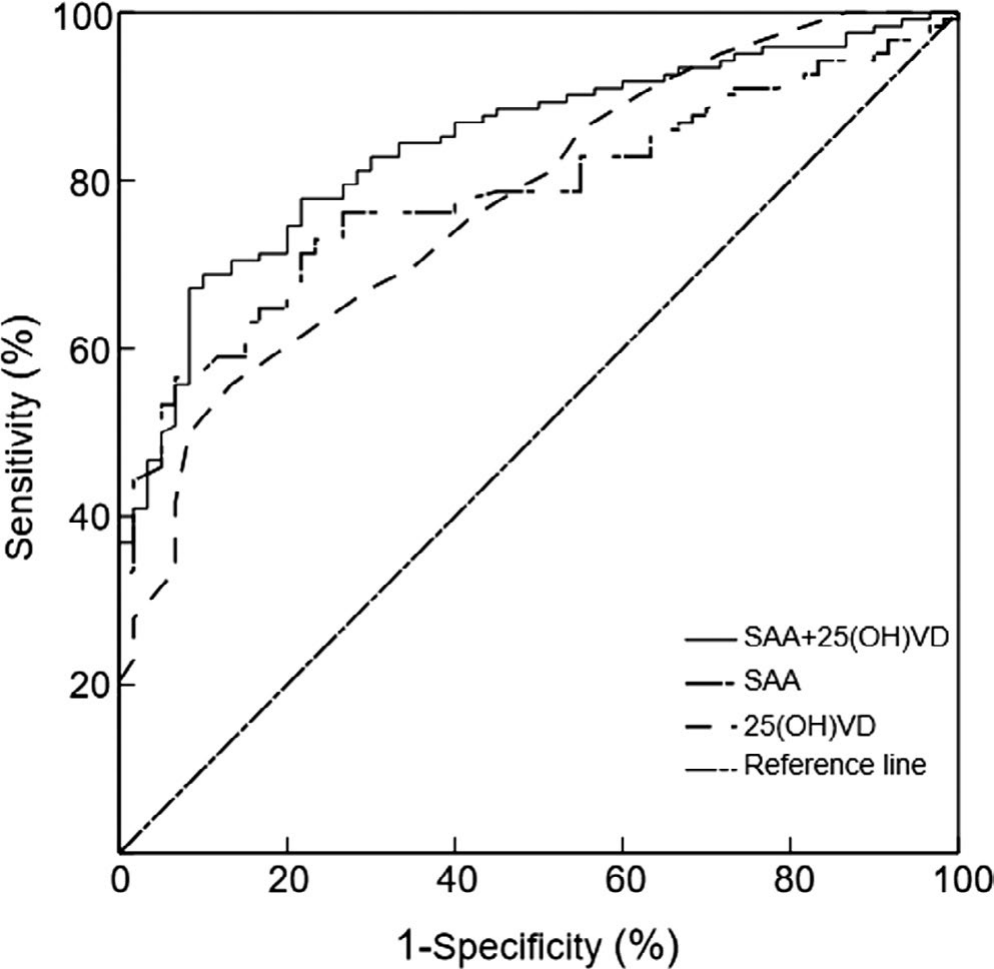
ROC curve of SAA and 25(OH)VD among patients with DN. When SAA was detected alone, the area under the ROC curve was 0.784 (95% CI: 0.720–0.849; *p* < 0.0001; cutoff value: 18.47 mg/L). When 25(OH)VD was detected alone, the area under the ROC curve was 0.775 (95% CI: 0.707–0.844; *p* < 0.0001; cutoff value: 24.50 ng/ml). When combined detection was performed, the area under the ROC curve was 0.843 (95% CI: 0.787–0.900; *p* < 0.0001)

According to the American Diabetes Association guidelines,^18^ the MA and CP groups were defined as having DN (UACR ≥30 mg/g). The ROC curve analysis indicated that the combined measurement of SAA and 25(OH)VD distinguishes between patients with T2DM and patients with DN better than the detection of SAA or 25(OH)VD alone (*p* < 0.05, Figure 3).

### 3.4 Independent risk factors for DN

Group allocation (NA, MA, and CP groups) was the dependent variable, and age, sex, BMI, SBP, DBP, smoking status, Urea, Crea, TC, TG, HDL‐C, LDL‐C, FBG, HbA1c, eGFR, SAA and 25(OH)VD were the independent variables. Binary logistic regression analysis showed that for each 1 mg/L increase in SAA, the risk of T2DM progressing to DN increased significantly (OR = 1.133, *p* = 0.000). The study also indicated that for each 1 ng/ml decrease in 25(OH)VD, the risk of T2DM progressing to DN increased significantly (OR = 0.838, *p* = 0.001). Therefore, our study demonstrated that the SAA level was an independent risk factor for DN, but the level of 25(OH)VD was an independent protective factor for DN (Table 3).

**TABLE 3:** Binary regression analysis of risk factors for diabetic nephropathy

Independent variable	*B*	SE	Wals *χ* ^2^	*p*‐value	OR (95% CI)
Age (years)	0.051	0.036	2.006	0.157	1.053 (0.980–1.131)
Sex (Females/males)	−1.493	0.847	3.106	0.078	0.225 (0.043–1.182)
BMI (kg/m^2^)	0.061	0.151	0.164	0.686	1.063 (0.790–1.430)
SBP (mmHg)	−0.043	0.024	3.168	0.074	0.958 (0.914–1.004)
DBP (mmHg)	0.050	0.063	0.615	0.433	1.051 (0.928–1.189)
Smoking status (yes/no)	−1.036	0.748	1.915	0.166	0.355 (0.082–1.539)
Urea (mmol/L)	−0.067	0.141	0.223	0.636	0.935 (0.709–1.234)
Crea (µmol/L)	0.036	0.019	3.768	0.052	1.037 (1.000–1.076)
TC (mmol/L)	0.284	0.600	0.225	0.636	1.329 (0.410–4.303)
TG (mmol/L)	0.526	0.623	0.712	0.399	1.692 (0.499–5.741)
HDL‐C (mmol/L)	−2.711	2.636	1.058	0.304	0.066 (0.000–11.649)
LDL‐C (mmol/L)	−0.841	0.771	1.189	0.275	0.431 (0.095–1.955)
FBG (mmol/L)	0.207	0.112	3.428	0.064	1.230 (0.988–1.532)
HbA1c (%)	0.546	0.322	2.879	0.090	1.727 (0.919–3.245)
eGFR (ml/min/1.73 m^2^)	−0.064	0.033	3.787	0.052	0.938 (0.880–1.000)
SAA (mg/L)	0.125	0.031	16.116	0.000	1.133 (1.066–1.204)
25(OH)VD (ng/ml)	−0.177	0.055	10.381	0.001	0.838 (0.753–0.933)
Constant	−2.776	7.928	0.123	0.726	0.062

Note

Binary logistic regression analysis was used to evaluate the risk factors of DN. *p*‐values <0.05 were considered statistically significant.

Abbreviations: 25(OH)VD, 25‐hydroxyvitamin D; B, regression coefficients; BMI, body mass index; CI, confidence interval; Crea, creatinine; DBP, diastolic blood pressure; eGFR, estimated glomerular filtration rate; FBG, fasting blood glucose; HbA1c, glycosylated hemoglobin A1c; HDL‐C, high‐density lipoprotein cholesterol; LDL‐C, low‐density lipoprotein cholesterol; OR, odds ratio; SAA, amyloid A; SBP, systolic blood pressure; SE, standard error; TC, total cholesterol; TG, triglycerides; Urea, urea.

## 4 DISCUSSION

Patients with T2DM have a high risk of cardiovascular events, and the occurrence and progression of DN increase the risk of death from cardiovascular diseases.^21^ Inflammation is the basis of all stages of DN and is core to the pathophysiology of DN and atherosclerosis.^22^ Previous studies have separately reported the association between SAA and diabetic microvascular complications or 25(OH)VD and diabetic microvascular complications.^23^, ^24^ Therefore, we combined the two indicators of SAA and 25(OH)VD to explore the combination's association with DN in the Chinese population. In this study, we found that both SAA and 25(O)VD levels are closely related to the occurrence and progression of T2DM. Compared with healthy individuals, T2DM patients have a significantly increased SAA level, while the level of 25(O)VD exhibited the opposite trend. Anderberg et al.^25^ found that SAA was significantly increased in the blood and was produced in the kidneys of both patients with diabetic kidney disease and diabetic mouse models. In addition, a longitudinal cohort study conducted by Dieter et al.^26^ indicated that higher levels of SAA could predict a higher risk of death and end‐stage renal disease in patients with T2DM. Lim et al.^27^ also showed that lower serum 25(OH)VD was related to poorer glycemic control and higher insulin use among multiethnic Asian patients with T2DM and stage 3–4 chronic kidney disease. A recent study conducted by Hong et al.^28^ also demonstrated that 25(OH)VD deficiency was common among patients with T2DM in Korea, and its mean level was 16.8 ± 9.6 ng/ml. The results of previous studies and this study have fully confirmed that SAA and 25(OH)VD are significantly related to the occurrence and severity of T2DM.

According to the level of UACR, T2DM patients were divided into three groups: T2DM with normal albuminuria, T2DM with microalbumin and T2DM with clinical albuminuria. We found that the SAA level of DN patients was significantly higher than that of diabetic patients with normal albuminuria and significantly increased along with levels of albuminuria. A study conducted by Dalla et al.^29^ indicated that acute‐phase markers (SAA, IL‐6, etc.) of inflammation were associated with DN; the level of SAA was significantly different among normoalbuminuric, microalbuminuric and proteinuric patients; and the SAA level was the highest in proteinuric patients. Kumon et al.^30^ also reported that SAA levels in patients with noninsulin‐dependent diabetes mellitus were significantly higher than those in healthy individuals and concluded that SAA seemed to be related to the development of DN. Both previous reports and our study can indicate that inflammation in the body is one of the important mechanisms that promotes the progression to DN in diabetic patients, and its specific regulatory mechanism needs in‐depth study. In addition, our study found that the serum 25(OH)VD level of patients with microalbuminuria and clinical albuminuria decreased significantly compared with that in T2DM patients with normal albuminuria and gradually decreased with the degree of kidney damage. An interesting study conducted by Ucak et al.^31^ showed that the level of microalbuminuria was significantly higher in T2DM patients with 25(OH)VD deficiency (≤10 ng/ml) than in patients with 25(OH)VD insufficiency (10–30 ng/ml). Another study also demonstrated that serum 25(OH)VD was significantly lower in T2DM patients than in healthy subjects, and the 25(OH)VD level in patients with DN was significantly lower than that in patients with diabetes without complications.^32^ Previous studies are consistent with our study, and all strongly indicated that 25(OH)VD deficiency is more common in patients with diabetes and plays an important role in the occurrence and progression of DN.

In the present study, we also found that combined measurement of SAA and 25(OH)VD can significantly improve the distinguishing of patients with DN (UACR ≥30 mg/g) from those with T2DM (UACR <30 mg/g). Furthermore, we explored the risk factors for DN by using binary logistic regression analysis and found that T2DM patients with higher levels of SAA might have a greater risk of developing DN. However, serum 25(OH)VD was an independent protective factor for DN, which means that high levels of serum 25(OH)VD may prevent T2DM patients from developing DN. A previous study reported by Dieter et al.^15^ based on a mouse model of diabetes showed that SAA (as a potent inflammatory mediator) was present throughout the diabetic kidney, and podocyte Janus kinase 2 overexpression increased tubulointerstitial SAA compared to the level observed in wild‐type diabetic controls. In addition, the research conducted by Xie et al.^33^ reported that the prevalence of 25(OH)VD insufficiency in T2DM patients with microalbuminuria was significantly higher than that in T2DM patients with normoalbuminuria, and low 25(OH)VD levels were associated with DN. Zhao et al.^34^ also demonstrated that 25(OH)VD was confirmed to be an independent protective factor for DN (OR = 0.962), which also means that 25(OH)VD deficiency is independently associated with a higher risk of DN. The above results were very consistent with the present study, and all fully confirmed that SAA promotes kidney function damage in diabetic patients by upregulating the expression of certain inflammatory factors. In addition, we can roughly speculate that serum 25(OH)VD may partially protect against the occurrence and development of DN by inhibiting inflammation, inhibiting oxidative stress, regulating immunity, and/or other mechanisms.

The present study had certain limitations. First, this was a cross‐sectional study, and we lacked long‐term follow‐up data to evaluate SAA and 25(OH)VD in patients with DN, so we cannot further clarify the continuous changes in the above indicators in the occurrence and progression of DN. Second, some potential confounding factors, such as sun exposure, nutritional status, season of 25(OH)VD detection, and potential inflammatory reactions, were not included in the analysis. Therefore, large‐scale and prospective cohort studies are required to clarify the causal relationships between SAA, 25(OH)VD and DN and the regulatory mechanisms in the occurrence and development of DN.

In conclusion, our study shows that patients with T2DM had higher SAA and lower 25(OH)VD and that the measurement of SAA and 25(OH)VD might be used to identify patients at increased risk of developing DN. However, many more prospective large‐scale trials are necessary to elucidate the regulatory mechanisms between SAA, 25(OH)VD and DN.

## CONFLICTS OF INTEREST

The authors declare no potential conflicts of interest with respect to the study, authorship, and/or publication of this article.

## AUTHOR CONTRIBUTIONS

QL and JS researched the literature and conceived the experiments. TDX and GRB were involved in protocol development, ethical approval, patient recruitment, and data analysis. QL, JS, and FMY wrote the first draft of the manuscript. All authors reviewed and edited the manuscript and approved the final version of the manuscript.

## Data Availability

The data are available upon request from the corresponding author (Fumeng Yang).
